# Trends in comorbidities and risk factors of patients with first time acute myocardial infarction between 1985 and 2023 – Results of the Myocardial Infarction Registry Augsburg

**DOI:** 10.25646/14266

**Published:** 2026-07-15

**Authors:** Timo Schmitz, Inge Kirchberger, Philip Raake, Jakob Linseisen, Christa Meisinger

**Affiliations:** 1 Epidemiology, Faculty of Medicine, University of Augsburg, Augsburg, Germany; 2 Department of Cardiology, Respiratory Medicine and Intensive Care, University Hospital Augsburg, Augsburg, Germany

**Keywords:** Body Mass Index, Coronary artery disease, Hyperlipidaemias, Prevalence, Myocardial infarction, Diabetes mellitus, Hypertension, Risk factors, Emergency medical services, Registries, Obesity, Smoking

## Abstract

**Background:**

For the last 40 years, the Myocardial Infarction Registry Augsburg recorded all cases of hospitalized acute myocardial infarction (AMI) and prehospital coronary artery disease (CAD) deaths. Obesity, hypertension, hyperlipidemia, diabetes mellitus and smoking are important risk factors for CAD and AMI.

**Methods:**

We analysed the presence and trends over time of these comorbidities in hospitalized patients with first-time AMI.

**Results:**

The prevalence of obesity (BMI > 30 kg/m²) in male and female AMI patients increased steadily from 1985 onwards until today. For hypertension and hyperlipidemia, there was an increase from 1985 until the early 2000s followed by a subsequent decline until 2023. Similarly, after an increase in the first two decades, the prevalence of diabetes in male AMI patients decreased slightly in recent years. For women, the proportion of patients with known diabetes remained unchanged in the first two decades but decreased since the turn of the millennium. While the number of male AMI patients that were current or former smokers did not vary much over time, there was a substantial increase in female patients.

**Conclusion:**

The prevalence of important comorbidities in hospitalized AMI patients changed within the past four decades. Especially the steady increase in obesity and smoking in female patients clearly shows that prevention efforts must be intensified to reduce the negative effects not only on coronary but also on cardiovascular and overall health.

**Key messages**

► Obesity has steadily increased over the past 40 years in male and female acute myocardial infarction (AMI) patients.
► The prevalence of hypertension and hyperlipidaemia peaked around the turn of the millennium and declined until afterwards.
► For men and women there was a slight decrease in the frequency of diabetes in recent years.
► Smoking habits of male AMI patients showed no major changes over the past 40 years.
► In female patients, the proportion of current or former smokers increased steadily from 1985 until nowadays.

## 1. Introduction

Worldwide, nine million people died in 2021 due to coronary artery disease (CAD) or its complications based on data from the World Health Organization [1]. According to official statistics (Federal Statistical Office of Germany), in Germany, one of three person died from cardiovascular diseases in 2024 [2]; and thereof every third person died from CAD or acute myocardial infarction (AMI) [3], which impressively demonstrates a major burden to public health due to CAD and AMI. Even though the number of cases and age-standardized mortality have declined in recent decades [4], these numbers impressively illustrate the importance of this disease for the majority of the population in Western countries. In recent decades, various risk factors have been identified that promote the development of CAD and are associated with a poorer prognosis after AMI. The most important behavioural risk factors include obesity and smoking [5]. However, comorbidities such as diabetes mellitus [6, 7], arterial hypertension [8] and elevated blood lipids, especially elevated LDL cholesterol [9], also promote the development of CAD and have a negative impact on the course of the disease. Knowledge of the development of these risk factors in AMI patients is crucial for specific preventive measures. Since 1985, the population-based Myocardial Infarction Registry Augsburg has been recording all cases of hospitalized AMI and coronary deaths in the study region. The basic methodology and specific methods of data collection and management have remained unchanged ever since which allows to investigate important time trends. The aim of this study was therefore to display and analyse the frequencies and time trends of major risk factors and comorbidities in hospitalized AMI patients based on data from the Myocardial Infarction Registry Augsburg and compare the developments in this specific group of patients with trends in the general population.

## 2. Methode

### 2.1 Study population

The Myocardial Infarction Registry Augsburg aims to capture all cases of hospitalized AMI and prehospital CAD deaths using a population-based approach. The study region consists of approximately 700,000 inhabitants (city of Augsburg and the two adjacent counties) [10]. All cases of hospitalized AMI and prehospital coronary deaths are included in case the patients’ age is 25 years or older and his or her primary residence is within the study region. Until 2008, only patients aged 25 – 74 years were recorded and from 2009 until 2018, also patients up to 84 years were considered. Since 2019, the upper age limit was removed to adapt to demographic changes and an aging society. 

A hospitalized AMI was defined as either ST-elevation myocardial infarction (STEMI), non-ST-elevation myocardial infarction (NSTEMI), or cases presenting with symptoms of unstable angina pectoris accompanied by elevated cardiac enzyme levels. For every case of hospitalized AMI a comprehensive bedside interview is conducted by a specially trained study nurse. Moreover, additional data is collected by extracting predefined information from the patient’s medical chart. In this way, a wide range of information is collected for each patient, including sociodemographic information, data on the acute event, symptoms, existing comorbidities, risk factors, treatment, medication (including medication before the event) and course during the hospital stay. 

Detailed information on methodology and data collection can be found in a prior publication [11]. All study participants gave written informed consent. Methods of data collection and questionnaires have been approved by the ethics committee of the Bavarian Medical Association (Bayerische Landesärztekammer), and the study was performed in accordance with the Declaration of Helsinki. The study was registered at the German Register of Clinical Studies (DRKS, project number DRKS00029042).

#### Comorbidities and risk factors in hospitalized AMI patients

In this study, we analysed the prevalence of important comorbidities and risk factors, namely obesity, diabetes mellitus, hypertension, hyperlipidaemia and smoking in hospitalized patients with a first-time AMI. The information on diabetes, hypertension and hyperlipidaemia was derived from the bedside interview as well as from the medical chart. If there was an indication of an existing condition (e.g. diabetes mellitus) in either the interview and/or the medical chart, the patient was regarded as having the respective preexisting condition at the time of the acute event. No distinction was made between recently diagnosed or long-treated comorbidity. Moreover, family doctors were not contacted to validate and complete the data on comorbidities. Information on smoking status (current smoker, former smoker, never smoker) was only taken from the bedside interview. Obesity was defined as a body mass index (BMI) of more than 30 kg/m², whereby weight was measured at the time of hospital discharge.

### 2.2 Statistical analysis

Categorical variables were presented as absolute frequencies with percentages, and Chi-square tests were applied to test for significant differences. For continuous variables, the results were presented as means with standard deviations and group differences were tested using analysis of variance (ANOVA).

The prevalence of existing comorbidities and risk factors are displayed as proportion (%) of all cases of first-time hospitalized AMI, whereby only patients aged 25 – 74 years were considered. Cases with missing values were not included in the analysis. Standard errors (SE) and 95 % confidence intervals (CI) were calculated using the normal approximation of the binomial distribution.

To display relevant changes over time and to smooth out statistical fluctuations, patients were grouped into 5-year intervals (the last interval is only a 4-year interval from 2020 to 2023). To maintain clarity, the baseline characteristics were displayed only for three of those 5-year intervals (the first, one in the middle, and the last). 

All analyses were performed using R version 4.4.3.

## 3. Results 

### 3.1 Baseline characteristics

A total of 16,848 hospitalized patients with a first-time AMI were included in this study. One quarter of all included patients were female (N = 4,208, male: N =12,640). The mean age was 63.2 (8.9) in female patients and 59.4 (9.6) years in male patients (see Table 1 and Table 2). In both sexes, close to half of all cases were ST-elevation myocardial infarctions (STEMI). The vast majority of male (87.3 %) and female patients (86.4 %) reported typical chest pain symptoms during the acute event. Over the past four decades, the level of education increased in men and women, although men are currently still more likely to have attained ‘Abitur’ or an equivalent education qualification. 

### 3.2 Comorbidities and risk factors 

**Figure 1:**
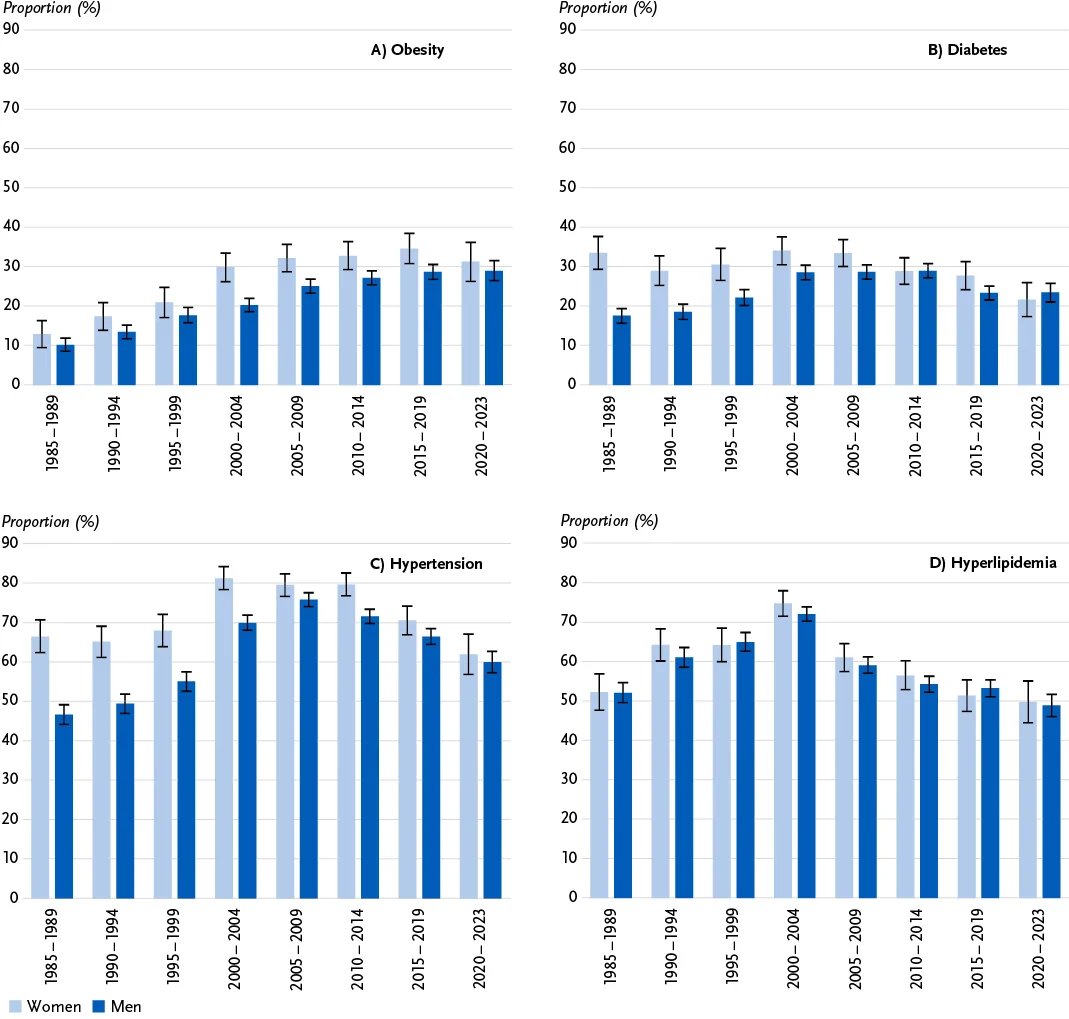
Prevalence of obesity (BMI > 30kg/m²), diabetes mellitus, hypertension and hyperlipidaemia in hospitalized AMI patients aged 25 – 74 years over time and stratified for sex (female N = 4,208, male N =12,640)

Over the last 40 years, prevalence of obesity substantially increased in both sexes (see Figure 1A and Supplementary material Table 1). While only 12.8 % (female) and 10.1 % (male) of all patients had BMI values of more than 30 kg/m² in 1985 –1989, the numbers increased to 31.2 % and 28.9 %, respectively, in 2020 – 2023. 

In female patients, the prevalence of diabetes mellitus remained stable between 1985 and 2009 with approximately 30 % of all patients diagnosed with this condition (Figure 1B and Supplementary material Table 1). After 2009, the numbers decreased to only 21.6 % in 2020 – 2023. In male patients, there was in increase between 1985 and the 2010s followed by a subsequent decrease (23.4 % in 2020 – 2023). The decline in diabetes prevalence is reflected by a slight decrease in diabetes treatment (drugs and/or insulin) in female patients, see Table 1. In men on the other hand, the proportion of patients receiving diabetes treatment rose during the past two decades.

The proportion of patients with hypertension rose between 1985 and the turn of the millennium, in men more pronounced than in women (see Figure 1C and Supplementary material Table 1). Subsequently, the numbers slightly decreased. In 2020 – 2023 around 60 % of hospitalized patients were diagnosed with hypertension before first-time AMI. In men and women, the frequency of antihypertensive medication (beta blocker, calcium channel blockers, diuretics) showed an overall decreasing trend, with the only exception of ACE inhibitor/Angiotensin II receptor blockers, see Table 1. 

A very similar trend as for hypertension was observed for hyperlipidemia, whereby almost identical numbers were found for female and male patients (see Figure 1D and Supplementary material Table 1). While about half of the patients had hyperlipidemia in 1985 –1989, the numbers increased to almost three-quarters in 2000 – 2004, followed by a steady decline back to about 50 % in 2020 – 2023. However, only a minor proportion of these patients took lipid lowering drugs before their first-time AMI, showing a small incline in frequency over the past 20 years especially in men, see Table 1. 

**Figure 2:**
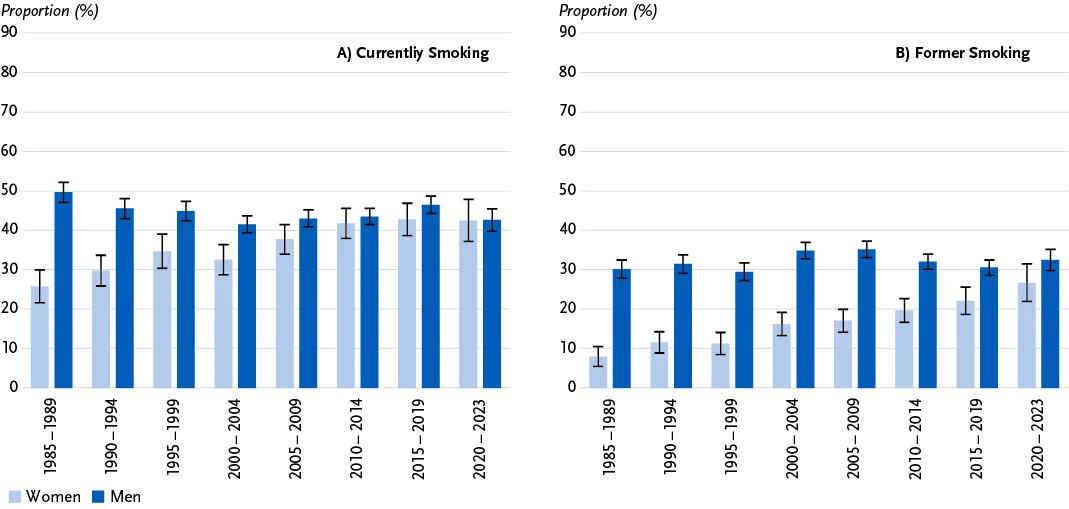
Smoking status of hospitalized AMI patients aged 25 – 74 years over time and stratified for sex (female N = 4,208, male N =12,640)

Smoking status of male patients remained roughly stable over time with about 40 – 50 % of patients being current smokers at the time of the acute event and another 30 – 35 % being former smokers (Figure 2A, Figure 2B and Supplementary material Table 1). In female patients, both, the proportion of current smokers as well as former smoker increased steadily over the past four decades, whereas in 2020 – 2023 42.4 % were currently smoking and 26.6 % were former smokers, (Figure 2A, Figure 2B and Supplementary material Table 1).

## 4. Discussion

### Baseline characteristics 

The analyzed study sample included patients with a first-time hospitalized AMI in the study region of Augsburg. Female patients were older at their first-time events compared to men. Despite the older age of women, 28-day survival rates were almost identical in 2020 – 2023 between men and women (both approximately 94 %). Overall, survival rates have risen in the past decades, in women more than in men. The main reason for this is supposedly improvements in acute treatment, and especially the increasing frequency and advancing technology in Percutaneous Coronary Intervention (PCI). As our data demonstrates, men and women had very similar frequencies of typical chest pain symptoms (87 % vs. 86 %), which is remarkable, since it has been reported, that both sexes differ in other symptoms accompanying AMI and women are more likely to have additional symptoms compared to men [12]. It was also demonstrated by data from the Augsburg Myocardial Infarction Registry that the absence of typical chest pain symptoms is associated with higher short- and long-term mortality [13]. 

### Principle findings

There were some major changes over the past four decades regarding the prevalence of important behavioral risk factors and comorbidities in patients with a first-time hospitalized AMI. While the prevalence of obesity was constantly rising in both sexes, the proportion of patients with diabetes mellitus, hypertension and hyperlipidemia increased approximately until the turn of the millennium followed by a slight decrease until nowadays. The smoking habits of male patients did not change considerably over the last 40 years, but the numbers of current or former smokers steadily increased in female patients. 

### Obesity

The increasing obesity prevalence is a common phenomenon in most countries around the world [14]. The situation in Germany is no exception with a rising prevalence in the general population in recent decades [15], a trend that is reflected also in hospitalized AMI patients as demonstrated by our results. However, while the prevalence in the general population is estimated to be around 20 % in 2023 [15], the prevalence is considerably higher in first-time hospitalized AMI patients in the study region of Augsburg with around 30 % in both sexes. This once more confirms obesity to be a major risk factor for many diseases including CAD and AMI, but also metabolic diseases [16]. Among others, major reasons for this increase in BMI and obesity are lack of physical activity and poor diet [17]. Even though many preventive measures have been installed in Germany in the last years, the trend has not (yet) been reversed [15]. However, there might be a so-called obesity paradox which describes the circumstance that overweight and obese AMI patients have a more favorable outcome, e.g. lower mortality after AMI, compared to their normal-weight counterparts [18, 19]. It is speculated for example that higher BMI likely represent higher muscle mass and bigger energy reserves and consequently less frailty, especially in older patients, which might be a main reason for better outcomes compared to normal weight individuals [18]. Although the obesity paradox is supported by several study results, latest studies also question the existence of the obesity paradox [20].

### Diabetes mellitus

Diabetes mellitus is a major risk factor for arteriosclerotic processes and CAD. This is reflected in the circumstance that almost one in four patients with a first-time hospitalized AMI had a diagnosis of diabetes according to our data. This is substantially higher than the figures for the general adult population, which, depending on the source, are likely to be around 10 % [21] and slightly higher in men than in women [22]. The latter circumstance is not reflected in hospitalized AMI patients, as according to our data, women had equal or even higher percentages of known diabetes over the past decades. A major reason for this might be the older age of women when experiencing the first hospitalized AMI (diabetes prevalence is rising with age in the general population) [23]. Not to be neglected is the presumably high number of AMI patients with previously undiagnosed diabetes [24, 25]. In a previous analysis from the Augsburg Myocardial Infarction Registry, we reported that in patients with ST-elevation myocardial infarction between 2009 and 2014, around 30 % of individuals had pre-diagnosed diabetes mellitus. Furthermore, one quarter of patients had unknown prediabetes and about 4 % of the study sample had an undiagnosed diabetes mellitus [25]. In the general German population, it has been reported that the prevalence of undiagnosed diabetes has decreased between 1998 and 2011 with about 2 % of adults between 18 and 79 have an undiagnosed diabetes [26]. There is no data available to verify whether this overall trend also applies to the patient with a first-time AMI. 

Diabetes not only increases the risk of developing CAD, but also is a major risk factor for higher short- and long-term mortality and worse outcome after hospitalized AMI [27, 28]. From a previous analysis based on data from this registry, we know that a substantial number of AMI patients with diagnosed diabetes is not treated optimally [29]. There are several major risk factors for diabetes mellitus type 2 itself; one major factor is overweight/obesity [30], which might have contributed to the rising prevalence of diabetes in male AMI patients from 1985 until about 2010. The reasons for the subsequent decline, observed in male and female patients, remain unclear. One could speculate that better blood glucose control in individuals with diabetes has prevented or positively affected the development of CAD and therefore resulted in a decreased percentage of patients with diabetes amongst those with first-time hospitalized AMI. 

### Hypertension and hyperlipidemia

Both hypertension and hyperlipidemia, especially elevated blood cholesterol, are considered key factors in the development of vascular calcification and arteriosclerosis [31]. Like diabetes mellitus, both conditions are exacerbated by obesity [32, 33].

The prevalence of hypertension increases markedly with age: in the general German population, it is between 30 and 40 % for individuals aged 45 – 64 years (higher in men than in women), and around 65 % for those aged 65 years or older [34]. Compared to these figures, the prevalence among patients with first-time hospitalized AMI in our analysis is not substantially higher. However, definitions of arterial hypertension vary between studies and analyses, and the number of individuals with undiagnosed hypertension is likely high, both of which significantly affect comparability. Regarding hyperlipidemia, a comparison with frequencies in the general population is even more difficult, as there are several different but commonly used definitions of hyperlipidemia [35]. 

In the present study, we found an increase in prevalence of hypertension and hyperlipidemia between 1985 and the 2000s which might be explained in part by increased awareness on the part of the treating physicians as it became more and more clear that both conditions are major risk factors and patients benefit from early intervention and prevention, which is also reflected in current recommendations of the ESC guidelines [36]. And indeed, an increasing number of AMI patients nowadays receive antihypertensive medication or lipid-lowering drugs, even before a first-time diagnosis of hospitalized AMI [4]. The effects of this circumstance might also contribute to the decrease in prevalence of hypertension and hyperlipidemia in AMI patients over the last two decades. Moreover, there is supposedly a high number of undiagnosed patients with these two comorbidities in the general population [37 38], which means that the actual frequencies are likely to be underestimated. 

### Smoking habits

The detrimental effects of smoking on cardiovascular and general health are well analyzed, documented and proven time and time again [39–41]. Smoking accelerates the processes of vascular inflammation, atherogenesis and plaque formation [42, 43]. Many prevention campaigns have been carried out in the last decades, and the number of smokers declined substantially in the general German population [44]. The proportion of current smokers in the adult male population dropped from almost 40 % in 1998 to less than 30 % in 2015. The frequency of current smokers amongst adult women is overall lower and the decline between 1998 and 2015 was slightly lower compared to men (from close to 30 % down to 22 %) [44]. However, the positive developments in smoking habits have recently stagnated somewhat [15]. In AMI patients, the proportion of current smokers is more than 40 % at the moment, and thereby substantially higher compared to the general population, once again demonstrating smoking to be a major risk factor for CAD and AMI. While in 1985, the proportion of current smokers among hospitalized AMI patients was almost twice as high in men compared to women, this difference has completely disappeared by 2023, which is in contrast to the general German population, where a significant difference between women and men still persists. The negative effects of smoking on cardiovascular diseases come with a certain delay, which might be a major reason for the consistently high numbers of male (former) smokers and even increasing numbers of female smokers and former smokers amongst AMI patients. It can be speculated that the favorable changes in smoking habits seen in the general population may be reflected in the data of the Augsburg Myocardial Infarction Registry in the upcoming years.

### Public health perspective

As discussed above, the present analysis demonstrates substantial differences in risk factors profiles between patients with a first-time hospitalized AMI and the general population in Germany. The circumstance that the prevalence of all analyzed comorbidities is higher in AMI patients is consistent with their known association with the development of CAD and AMI. Although age-standardized AMI rates and mortality decreased over the past decades [4], the burden for public health in Germany and world-wide remains very high. As mentioned above, almost 120,000 people died due to AMI or CAD in Germany in 2023, which correlates to about 12 % of all deaths [3]. An aging society ensures that this disease will continue to be of paramount importance in the future health care. A major tool for reducing the burden of disease is and will be primary prevention, and importantly, proper medical treatment of AMI risk factors. As our data demonstrates, there have been some positive developments in the last two decades regarding diabetes, hypertension and hyperlipidemia in AMI patients. However, there are still important tasks ahead. Especially the still rising prevalence of obesity, which is also observed in the general population [15], requires an enhanced effort in general education and prevention. But also smoking habits among hospitalized AMI patients have not shown a favorable development, especially in female patients. The number of smokers in the general population has dropped in Germany over the last decades [44], a development not (yet) mirrored in hospitalized AMI patients. Consequently, the data reaffirms the importance of continued and consistent efforts in smoking prevention, especially with regard to cardiovascular health and coronary heart disease. 

### Strengths and limitations 

The major strength is the stable methodology and data collection of the Myocardial Infarction Registry Augsburg over more than 40 years. In combination with the population-based approach with complete enrollment in the study region, which minimizes selection bias, the data is best suited to describe trends over time. The process of data collection, including the interview, is standardized and carried out by trained study nurses. Since the study region includes urban and rural areas, it can be considered representative for the South German population. 

Nevertheless, some limitations need to be mentioned. Only patients aged 25 – 74 years were captured from 1985 until 2009, so for the elderly no long-term trends can be reported. Methods of data collection might be different from other registries around the world, which limits comparability with other countries and regions. No information on ethnicity was available, so results might not be applicable to all ethnic groups. Finally, the last period of data collection (2020 – 2023) falls into the Covid-19-pandemics. The latter not only complicated case identification and data collection but also affected the number of hospitalizations due to AMI. As a previous publication from this Registry demonstrated, the number of hospitalizations dropped during the first phase of lockdown dramatically [45]. Moreover, the decrease might haven been more pronounced in NSTEMI cases compared to STEMI cases in Germany [46], which could explain the high percentage of STEMI cases in the time period 2020 – 2023 in the present study. 

### Conclusion

The prevalence of obesity in hospitalized AMI patients constantly rose over the last four decades. The proportion of patients with diagnosed diabetes mellitus, hypertension or hyperlipidemia increased until the beginning of 2000s followed by a slight decrease. While smoking habits hardly changed among men, the numbers of current and former smokers increased in female patients. Overall, the prevalence of all analyzed risk factors is substantially higher in first-time AMI patients compared to the general population, although trends have developed differently in some cases over the past decades. These circumstances and developments indicate that continued efforts in primary prevention of behavioral risk factors and early detection and proper treatment of comorbidities may help to minimize the burden of disease caused by CAD and AMI.

## Figures and Tables

**Table 1:** Baseline characteristics of the female first-time AMI in-patients, total (N = 4.208) and stratified for three different time periods. Source: Myocardial Infarction Registry Augsburg

	**Total sample** **N = 4,208**			**1985 –1989** **N = 464**			**2000 – 2004** **N = 618**			**2020 – 2023** **N = 320**			**p-value**
	**Mean**	**SD**	N^*^	**Mean**	**SD**	**% Missing**	**Mean**	**SD**	**% Missing**	**Mean**	**SD**	**% Missing**	
Age at AMI^1^ (mean, SD)	63.2	8.9	4,208	64.5	7.8	0	63.7	8.7	0	62.4	8.5	0	0.003
	Absolut Frequencies	Percentages		Absolut Frequencies	Percentages		Absolut Frequencies	Percentages		Absolut Frequencies	Percentages		
**Sociodemographic information **													
Education			3,171			43.1			28.8			9.4	< 0.001
Hauptschule/Other (≤ 9 years)	2,369	74.7		224	84.8		364	82.7		166	57.2		
Realschule (10 –11 years)	598	18.9		34	12.9		59	13.4		85	29.3		
Abitur/Hochschulreife (12 –13 years)	204	6.4		6	2.3		17	3.9		39	13.4		
Married^**^	1,678	59.5	2,820	-	-	-	356	58.7	1.9	193	62.1	2.8	0.229
Currently employed/working^**^	536	25.7	2,083	-	-	-	103	18.0	7.6	115	38.5	6.6	< 0.001
**Acute event**													
Type of infarction			4,018			7.3			3.4			3.8	< 0.001
STEMI^2^	1,823	45.4		198	46.0		261	43.7		187	60.7		
NSTEMI^3^	1,958	48.7		232	54.0		336	56.3		121	39.3		
Presence of typical chest pain symptoms	3,587	86.4	4,150	366	81.3	3.0	539	87.9	0.8	267	86.1	3.1	0.010
Survived the first 28 days	3,874	92.1	4,208	407	87.7	0.0	569	92.1	0	301	94.1	0.0	0.005
**Comorbidities and risk factors**													
Obesity (BMI^4^ > 30 kg/m^2^)	1,024	27.7	3,702	41	12.8	31.0	162	29.8	12.0	96	31.2	3.8	< 0.001
Diabetes mellitus	1,270	30.3	4,190	151	33.5	2.8	210	34.0	0.0	69	21.6	0.0	< 0.001
Hypertension	3,051	72.9	4,188	299	66.4	3.0	501	81.2	0.2	198	61.9	0.0	< 0.001
Hyperlipidemia	2,482	60.0	4,135	217	52.2	10.3	461	74.7	0.2	159	49.7	0.0	< 0.001
Smoking			3,752			17.9			16.8			5.0	< 0.001
Currently smoker	1,355	36.1		98	25.7		167	32.5		129	42.4		
Ex smoker	619	16.5		30	7.9		83	16.1		81	26.6		
Never smoker	1,778	47.4		253	66.4		264	51.4		94	30.9		
	Absolut Frequencies	Percentages		Absolut Frequencies	Percentages		Absolut Frequencies	Percentages		Absolut Frequencies	Percentages		
**Medication before first-time AMI**													
Beta blocker	913	27.1	3,371	62	14.8	9.5	200	34.6	6.5	75	24.4	4.1	< 0.001
ACE inhibitor/ARBs^5^	886	26.3	3,370	13	3.1	9.7	178	30.8	6.5	108	35.1	3.8	< 0.001
Calcium channel blockers	743	22.0	3,370	116	27.7	9.7	118	20.4	6.3	40	13.0	4.1	< 0.001
Diuretics	872	25.8	3,375	128	30.3	9.1	165	28.5	6.5	35	11.4	3.8	< 0.001
Lipid lowering drugs	463	13.6	3,407	18	4.2	8.2	86	14.7	5.3	47	15.3	4.1	< 0.001
Diabetes medication/Insulin^**^	452	15.6	2,889	-	-	-	97	15.7	0.0	39	12.2	0.0	0.272
**In-hospital treatment **													
PCI^6^	2,205	52.6	4,196	16	3.5	0.6	316	51.1	0.0	300	93.8	0.0	< 0.001
Bypass surgery	380	9.1	4,187	21	4.6	0.6	87	14.1	0.2	18	5.6	0.0	< 0.001
Lysis therapy	515	12.4	4,158	56	12.2	0.9	97	15.8	0.5	4	1.3	1.6	< 0.001

^*^ Number of cases with valid information

^**^This information was not collected in the first years of the Registry

^1^ Acute myocardial infarction

^2^ ST-elevation myocardial infarction

^3^ Non-ST-elevation myocardial infarction (including bundle branch block in the admission ECG)

^4^ Body mass index

^5^ Angiotensin II receptor blockers

^6^ Percutaneous Coronary Intervention

**Table 2:** Baseline characteristics of the male first-time AMI in-patients, total (N =12.640) and stratified for three different time periods. Source: Myocardial Infarction Registry Augsburg

	**Total sample** **N =12,640**			**1985 –1989** **N =1,370**			**2000 – 2004** **N =1,873**			**2020 – 2023** **N =1,084**			**p-value**
	**Mean**	**SD**	N^*^	**Mean**	**SD**	**% Missing**	**Mean**	**SD**	**% Missing**	**Mean**	**SD**	**% Missing**	
Age at AMI^1^ (mean, SD)	59.4	9.6	12,640	58.8	9.4	0	59.4	9.9	0	59.6	9.2	0	0.097
	Absolut Frequencies	Percentages		Absolut Frequencies	Percentages		Absolut Frequencies	Percentages		Absolut Frequencies	Percentages		
**Sociodemographic information**													
Education^**^			10,176			31.1			25.3			10.0	< 0.001
Hauptschule/Other (≤ 9 years)	6,754	66.4		718	76.1		1,020	72.9		508	52.0		
Realschule (10 –11 years)	1,606	15.8		148	15.7		174	12.4		210	21.5		
Abitur/Hochschulreife (12 –13 years)	1,816	17.8		78	8.3		206	14.7		258	26.4		
Married^**^	6,668	75.8	8,793	-	-	-	1,487	80.9	1.9	744	70.2	2.2	< 0.001
Currently employed/ working^**^	3,159	48.3	6,540	-	-	-	745	42.5	6.5	608	59.6	5.9	< 0.001
**Acute events**													
Type of infarction			12,065			7.7			3.7			4.6	< 0.001
STEMI^2^	5,613	46.5		570	45.1		809	44.9		576	55.7		
NSTEMI^3^	6,452	53.5		694	54.9		994	55.1		458	44.3		
Presence of typical chest pain symptoms	10,923	87.4	12,493	1,159	85.5	1.1	1,638	88.1	0.7	958	90.1	1.9	0.003
Survived the first 28 days	11,881	94.0	12,640	1,261	92.0	0.0	1,748	93.3	0.0	1,022	94.3	0.0	0.087
**Comorbidities and risk factors**													
Obesity (BMI^4^ > 30 kg/m^2^)	2,548	22.2	11,463	106	10.1	23.6	338	20.2	10.6	301	28.9	4.0	< 0.001
Diabetes mellitus	3,086	24.5	12,610	236	17.5	1.3	532	28.5	0.3	253	23.4	0.1	< 0.001
Hypertension	7,991	63.5	12,593	625	46.6	2.1	1,306	69.9	0.3	648	59.9	0.2	< 0.001
Hyperlipidemia	7,291	58.6	12,440	655	52.0	8.1	1,344	72.0	0.3	528	48.8	0.2	< 0.001
Smoking			11,804			8.1			11.7			4.6	< 0.001
Currently smoker	5,247	44.5		624	49.6		685	41.4		440	42.6		
Ex smoker	3,790	32.1		379	30.1		576	34.8		335	32.4		
Never smoker	2,767	23.4		256	20.3		392	23.7		259	25.0		
	Absolut Frequencies	Percentages		Absolut Frequencies	Percentages		Absolut Frequencies	Percentages		Absolut Frequencies	Percentages		
**Medication before first-time AMI**													
Beta blocker	1,948	19.4	10,032	131	10.5	9.1	391	22.7	8.1	179	17.3	4.5	< 0.001
ACE inhibitor/ARBs^5^	2,047	20.4	10,033	27	2.2	9.1	362	21.0	8.1	343	33.1	4.5	< 0.001
Calcium channel blockers	1,499	14.9	10,032	216	17.3	8.8	253	14.7	8.2	129	12.5	4.5	0.005
Diuretics	1,493	14.9	10,038	199	15.9	8.8	278	16.1	8.1	73	7.1	4.5	< 0.001
Lipid lowering drugs	1,245	12.3	10,143	67	5.3	8.2	220	12.6	6.5	171	16.5	4.4	< 0.001
Diabetes medication/Insulin^**^	1,196	13.4	8,952	-	-	-	223	11.9	0.1	161	14.9	0.0	0.032
**In-hospital treatment**													
PCI^6^	7,285	57.8	12,601	61	4.5	0.7	1,123	60.1	0.3	1,001	92.4	0.1	< 0.001
Bypass surgery	1,414	11.2	12,595	42	3.1	0.7	339	18.1	0.2	78	7.2	0.2	< 0.001
Lysis therapy	1,797	14.4	12,487	291	21.4	0.8	345	18.6	0.8	8	0.7	0.4	< 0.001

^*^ Number of cases with valid information

^**^This information was not collected at the start of the Registry

^1^ Acute myocardial infarction

^2^ ST-elevation myocardial infarction

^3^ Non-ST-elevation myocardial infarction (including bundle branch block in the admission ECG)

^4^ Body mass index

^5^ Angiotensin II receptor blockers

^6^ Percutaneous Coronary Intervention
